# Andiroba Oil (*Carapa guianensis* Aublet) Gel-Based Nanoemulsion: Development, Characterization, and In Vitro Evaluation in Wound Healing-Related Cells

**DOI:** 10.1021/acsomega.6c02635

**Published:** 2026-10-01

**Authors:** Vitória R. P. Silva, Atailson O. da Silva, Mosar C. Rodrigues, Karen R. Py-Daniel, Bruna R. B. Gomes, Elysa B. O. Damas, Thalya S. R. Nogueira, Ivo J. C. Vieira, Mariana S. Castro, Marcelo H. Sousa, Marcella L. B. Carneiro, Graziella A. Joanitti

**Affiliations:** † Laboratory of Bioactive Compounds and Nanobiotechnology (LBCNano), 28127 University of Brasilia (UnB), Campus Darcy Ribeiro, Brasília, DF 70910-900, Brazil; ‡ Post-Graduation Program in Pharmaceutical Science, University of Brasilia (UnB), Campus Darcy Ribeiro, Brasília, DF 70910-900, Brazil; § Green Nanotechnology Group, University of Brasilia (UnB), Centro Metropolitano, Ceilandia Sul, Brasília, DF 72220-275, Brazil; ∥ Laboratory of Protein Chemistry and Biochemistry, Department of Cell Biology, Institute of Biological Sciences, 223023 University of Brasilia, Brasilia, DF 70910-900, Brazil; ⊥ Laboratory of Chemical Sciences (LCQUI), State University of Northern Fluminense Darcy Ribeiro (UENF), Avenida Alberto Lamego 2000, Campos dos Goytacazes, RJ 28013-602, Brazil; # Post-Graduation Program in Nanoscience and Nanobiotechnology, University of Brasilia (UnB), Campus Darcy Ribeiro, Brasília, DF 70910-900, Brazil

## Abstract

Andiroba oil (*Carapa guianensis* Aublet) is widely used in traditional medicine due to its anti-inflammatory and reported wound-healing properties. In this study, we developed and characterized andiroba oil-based nanoemulsions (NeAnd) and gel-based nanostructured formulation (NeAndG) to investigate their physicochemical stability and initial in vitro biological effects in wound healing-related cells. Chemical characterization by GC-MS confirmed the presence of limonoids (including gedunin and its derivatives), β-sitosterol, and oleic acid in andiroba oil composition, compounds associated with anti-inflammatory and regenerative properties. The nanoemulsions exhibited hydrodynamic diameters <200 nm, low polydispersity indices (<0.3), and a negative surface charge, indicating a homogeneous and stable nanostructured system. After incorporation into the Carbopol hydrogel, the nanodroplets maintained nanoscale size while exhibiting a markedly increased negative ζ potential (≈ −74 mV), suggesting enhanced colloidal stability. Long-term stability assays demonstrated that the gel matrix significantly improved thermal stability and prevented nanodroplet aggregation compared with the nanoemulsion alone during storage (4, 25, and 37 °C for 120 days). In vitro assays using human keratinocytes (HaCaT) and fibroblasts (HGF) showed a dose-dependent decrease in cell viability. Scratch assay analysis revealed no significant enhancement of cell migration compared with controls, suggesting that the biological activity of andiroba oil in this context may involve mechanisms other than cell migration. Overall, these results demonstrate that the andiroba oil nanogel represents a stable and biocompatible nanoplatform suitable for further investigation in wound healing-related studies.

## Graphical abstract

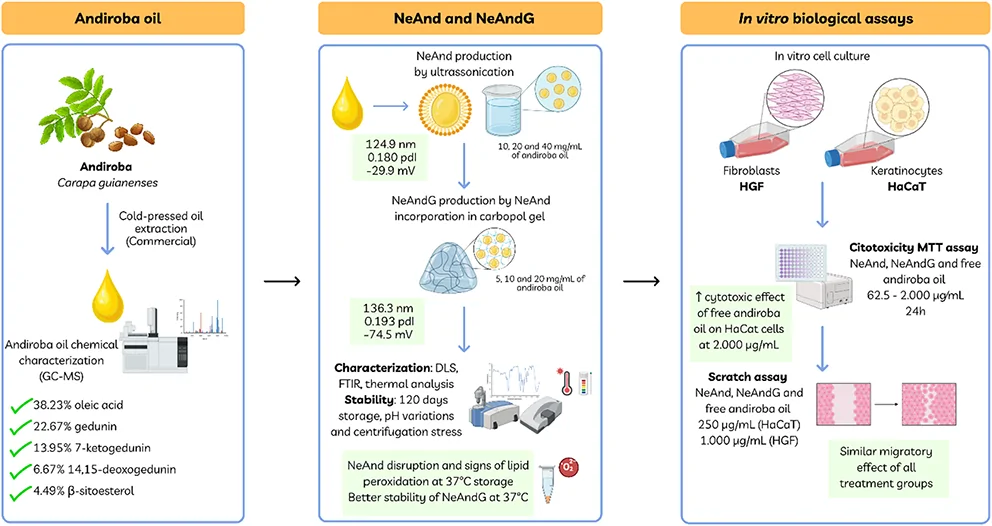

## 1 Introduction

The wound healing process involves the restoration of injured tissue and consists of four phases: hemostasis, inflammation, proliferation, and remodeling. The progression through these phases is dynamic, mediated by growth factors and pro- and anti-inflammatory molecules that regulate the activity of cells responsible for collagen degradation, synthesis, and stabilization.^1–3^ Synchronizing these healing processes is crucial for efficient tissue regeneration and rapid wound repair. Inefficiencies in the healing process are associated with various systemic pathologies, like diabetes mellitus.

Andiroba (*Carapa guianensis* Aublet) is a tree from the *Meliaceae* family found in tropical regions of South and Central America. In Brazil, it grows predominantly in the northern Amazon region.^4^ From the seeds of the andiroba tree, it is possible to extract an oil widely utilized in traditional medicine as an antipyretic, analgesic, anti-parasitic, anti-inflammatory, antiseptic, and healing agent.^4,5^ The extensive therapeutic potential of andiroba oil has positioned this natural product prominently for the development of therapeutic and cosmetic formulations, particularly due to its remarkable wound healing properties.

Quantitatively, andiroba oil comprises a higher proportion of fatty acids, mainly oleic acid and palmitic acid, and a small proportion of linoleic acid, and stearic acid.^6^ Additionally, it contains 3-5% secondary metabolites, including triterpenes, steroids, coumarins, flavonoids, and limonoids.^5^ These compounds play crucial roles in accelerating tissue healing by stimulating cell proliferation and collagen production, while also exhibiting antimicrobial, antioxidant, and anti-inflammatory effects.^7^

Incorporation of this oil into nanostructures presents an alternative for optimizing its interaction with the biological microenvironment and modulating its biological effects. Nanoemulsion systems, in this regard, are formulations capable of enhancing the healing activity by encapsulating it in nanometer-sized droplets, typically ranging from 100 to 300 nm, stabilized by a surfactant. The reduced size achieved increases the contact surface area of the oil with cells, facilitating greater interaction of bioactive molecules with their cellular targets and thereby potentially modifying their in vitro cellular response.^8^

Our research group has already developed a liquid andiroba oil-based nanoemulsion and reported its physicochemical features and initial in vitro biological effects.^9^ In this context, the present work involves the next step, which includes improvements in the nanoformulation development (*e.g.*, addition of co-surfactant, variation in oil concentrations, changes in processing parameters) as well as its incorporation into a gel base (hydrogel) to facilitate application to skin wounds and optimize its physicochemical stability. In the context of skin wounds, nanostructured gels display important physicochemical properties that aid in treatment. The gel acts as a diffusional barrier, supporting the sustained release of bioactive compounds at the application site, which enables prolonged therapeutic action and may decrease the frequency of applications.^10^ Therefore, the goal of this study was to develop an andiroba oil nanostructured gel and assess its cytotoxicity and effects on the migratory activity of wound healing-related cells (keratinocytes and fibroblasts) in vitro.

## 2 Materials and Methods

The following materials were acquired for preparing the formulations: ultrapure water (Milli-Q, Merck Group, Darmstadt, Germany), lecithin (Lipoid, Ludwigshafen, Germany), cold-pressed fixed andiroba oil (Ferquima, São Paulo, Brazil), polyoxyethylene (40) stearate (PEG 40) (Sigma-Aldrich Ltd., Gillingham, Dorset, UK), Carbopol 940 (Casa da Química Ltd., Brasília, Brazil), and triethanolamine (TEA) (Dinâmica Química Contemporânea Ltda., São Paulo, Brazil).

For cell culture, fetal bovine serum, Dulbecco’s modified Eagle’s medium (DMEM), trypsin, and streptomycin were purchased from Gibco Scientific (USA). 3-(4,5-dimethylthiazol-2-yl)-2,5-diphenyltetrazolium bromide (MTT) (Invitrogen, USA), and dimethyl sulfoxide (DMSO) (J.T.Baker, Phillipsburg, USA) were used. Human gingival fibroblasts were purchased from Rio de Janeiro Cell Bank (BCRJ, Rio de Janeiro, Brazil), and keratinocytes were donated by Professor Michel Fleith Otuki from the Federal University of Paraná (Paraná, Brazil).

### 2.1 Andiroba Fixed Oil Characterization

The andiroba oil was characterized, with a focus on detecting secondary metabolites. The presence of secondary metabolites in the oil was investigated using gas chromatography-mass spectrometry (GC-MS), as previously described by Ombredane and collaborators (2022), employing an Agilent 5975C mass selective detector coupled to a 7890A gas chromatograph.^11^ Compound identification was performed by comparing the obtained mass spectra with the NIST library database, using both the similarity index and visual inspection.

### 2.2 Production of Nanoemulsion and Its Incorporation into a Carbopol Gel Base

Lecithin and andiroba oil were weighed in a 2:1 ratio (w/w) to produce andiroba oil nanoemulsions (NeAnd) containing 10, 20, and 40 mg/mL of andiroba oil. PEG 40 stearate at 1% was added relative to the molar mass of lecithin, along with 10 mL of ultrapure type I water. The mixture was subjected to ultrasonication in an ice bath for 10 min with alternating pulses (1 second each) at 20 kHz. Blank formulation was prepared without the addition of andiroba oil. The production of nanogels containing andiroba oil nanoemulsions (NeAndG) was performed according to the protocol of Jangdey, and collaborators (2017) with modifications.^12^ First, a Carbopol gel base was prepared by diluting 0.5% (w/v) of Carbopol 940 in ultrapure water with manual stirring. The solution was stored for 2 h at room temperature (25 °C), and triethanolamine was used to adjust the pH to approximately 7.0. The NeAnd at 10, 20, and 40 mg/mL (NeAnd 10, 20, and 40, respectively) was incorporated into the Carbopol 940 by gentle manual stirring, forming NeAndG at 5, 10, and 20 mg/mL (NeAndG 5, 10, and 20, respectively), which was stored protected from light at 4 °C.

### 2.3 Dynamic Light Scattering (DLS), Electrophoretic Light Scattering (ELS) and pH

The ZetaSizer lab equipment (Malvern, UK) was used to measure the hydrodynamic diameter, polydispersity index (PdI), and ζ potential of NeAnd and NeAndG. NeAnd formulations were diluted at a 1:100 (v/v) ratio (10 μL of the sample and 990 μL of deionized water), and 10 mg of NeAndG was weighed with an analytical balance and diluted in 990 μL of deionized water. The pH of the formulations diluted at a 1:100 (v/v) ratio in deionized water was 7. Each sample was measured in triplicate at 25 °C with a scattering angle of 90°. The pH of the formulations was measured using a universal pH strip (Kasvi, BR).

### 2.4 FTIR Analysis

Fourier transform infrared spectroscopy (FTIR) measurements were performed on an Agilent Cary 630 spectrometer coupled to a Diamond ATR accessory module (Agilent, US); scans were performed from 4000 to 650 cm^–1^ (absorption mode) with readings of 96 scans recorded at 2 cm^–1^ resolution per sample. NeAnd 10 mg/mL, NeAndG 5 mg/mL, and its isolated components (Free andiroba oil, Lecithin, and Carbopol Gel) were analyzed.

### 2.5 Thermal Analysis

Thermogravimetric analysis (TGA) and differential thermal analysis (DTA) were performed using a Shimadzu DTG-60 instrument (Shimadzu, Japan) with nitrogen as the carrier gas. Samples were heated at a rate of 25 °C/min, and data were collected from 35 to 1000 °C. Analyses were conducted for NeAnd 10 mg/mL, NeAndG 5 mg/mL, and Carbopol gel.

### 2.6 Stability of the Andiroba Gel-Based Nanoemulsion as a Function of pH

The pH adjustments of NeAnd 10 mg/mL and NeAndG 5 mg/mL were performed using an automatic MPT-2 autotitrator (Malvern, UK) with NaOH and/or HNO_3_ standard solutions as titrants to achieve pH levels of 3, 5, 7, 9, and 11. DLS and ELS analysis (as described in Section 2.3) were performed.

### 2.7 Accelerated Stability

Two aliquots of 1.0 mL of NeAnd at 10, 20, and 40 mg/mL, and NeAndG at 5, 10, and 20 mg/mL, were centrifuged at 9.500 RCF for 30 min at 25 °C using a Hettich Universal 320R centrifuge (Tuttlingen, Germany). DLS and ELS analysis (as described in 2.3) were performed before and after centrifugation.

### 2.8 Long-Term Thermal Stability

The samples were stored for 120 days at temperatures of 4, 25, and 37 °C, with protection from light. The stability of NeAnd 10, 20, and 40 mg/mL, and NeAndG 5, 10, and 20 mg/mL was evaluated by measuring hydrodynamic diameter, PdI, pH, and ζ potential as described in Section 2.3. Measurements were taken on days 0, 15, 30, 60, 90, and 120.

### 2.9 Cell Culture

Human non-cancerous keratinocytes (HaCaT) and human fibroblasts (HGF) were cultured in DMEM medium supplemented with 10% (v/v) fetal bovine serum and 1% (v/v) penicillin-streptomycin. The cells were incubated at 37 °C with 5% CO_2_. Experiments were conducted on cells at passages 3 to 8. All experiments were carried out in experimental triplicate. The interval between formulation synthesis and their use in *in vitro* tests was up to 2 weeks.

### 2.10 Cell Viability Assay

Cells were seeded in 96-well plates at a density of 1.0 × 10^4^ cells/well for fibroblasts and 5.0 × 10^3^ cells/well for HaCaT cells, then maintained in a humidified incubator at 37 °C with 5% CO_2_. After 24 h, the cells were treated with andiroba-based nanoemulsion (NeAnd) and gel form (NeAndG), free andiroba oil (OL), and its gel form (OLG), using the following oil concentrations: 62.5, 125, 250, 500, 1000, and 2000 μg/mL. Cells were also treated with the Carbopol gel base alone (gel), and the blank formulation without andiroba oil (Br) and its gel form (BrG). To prepare the treatments, the gel formulations were weighed on an analytical balance and diluted in culture medium to reach the desired test concentrations. Free andiroba oil was diluted in ethanol, with the final ethanol concentration in the culture media being 1%. A control group treated with 1% ethanol was also included. After 24 h of incubation the treatments were discarded, and each well received 150 μL of MTT solution at 0.5 mg/mL diluted in DMEM. After 1 h at 37 °C, the MTT solution was removed, and the formed formazan crystals were dissolved with 100 μL of dimethyl sulfoxide (DMSO) in each well. Absorbance was measured with a spectrophotometer (M Varioskan LUX Multimode Microplate Reader, USA) at 595 nm.

### 2.11 Scratch Assay

The migratory ability of keratinocytes and fibroblasts was evaluated using the scratch assay. A 24-well plate was prepared by placing a culture insert (Ibidi, Munich, Germany) into each well with sterile tweezers, pressing the edges of the inserts to ensure they adhered to the plate surface. Next, 7 × 10^4^ HaCaT cells (human keratinocytes) or 2.5 × 10^4^ HGF cells (human fibroblasts) in 70 μL of Dulbecco’s Modified Eagle’s Medium (DMEM), supplemented with 10% fetal bovine serum (FBS) and 1% antibiotic solution, were pipetted into each compartment of the culture insert. The plate was then incubated at 37 °C with 5% CO_2_ for 24 h to form a confluent cell monolayer. After 24 h of incubation, the inserts were carefully removed. Then, DMEM with 1% FBS was added to each well. The cell-free gaps were visualized under an EVOS microscope at 10 × magnification. Next, cells were incubated for 24 h with andiroba-based nanoemulsion (NeAnd) and gel form (NeAndG), Carbopol (gel), free andiroba oil (OL), and gel form (OLG), as well as blank controls (Br and BrG), at a concentration of 250 μg/mL for keratinocytes and 1,000 μg/mL for fibroblasts in DMEM with 1% FBS. Images of each gap were captured before treatment (T0) and after 24 h (T24). The total wound area in each well was quantified using ImageJ software, following the method described by Venter and Niesler (2019).^13^ The percentage of wound closure was calculated with the equation: % Wound Closure = ((Wound area at 0 h – Wound area at 24 h)/Wound area at 0 h) × 100.

### 2.12 Statistical Analysis

Statistical analysis was conducted by first applying the Shapiro-Wilk normality test. Parametric data were analyzed using one-way analysis of variance (ANOVA) for data with a single independent variable, followed by Dunnett’s post-test, and two-way ANOVA for data with two or more independent variables, followed by Tukey’s post-test. Nonparametric data were analyzed with the Friedman test followed by Dunn’s post-test for paired data, and the Kruskal-Wallis test followed by Dunn’s post-test for unpaired data. Analyses were performed using GraphPad Prism version 10.2.3 (GraphPad Software, San Diego, California, USA). Significant differences were considered when *p* < 0.05.

## 3 Results and Discussion

### 3.1 Chemical Characterization of Andiroba Oil

Our previous study described data regarding acidity index, peroxide value, lipid profile, carotenoid profile, total phenolic compounds, and antioxidant activity of andiroba oil.^9^ In the present study, the focus of the characterization was the detection of secondary metabolites. The method used allowed the identification of five long-chain hydrocarbons, whether saturated or unsaturated, with a carboxylic acid or aldehyde group, as well as three limonoids and one phytosterol, which are described in Table 1 and have their chemical structures presented in Figure 1.

**Figure 1:**
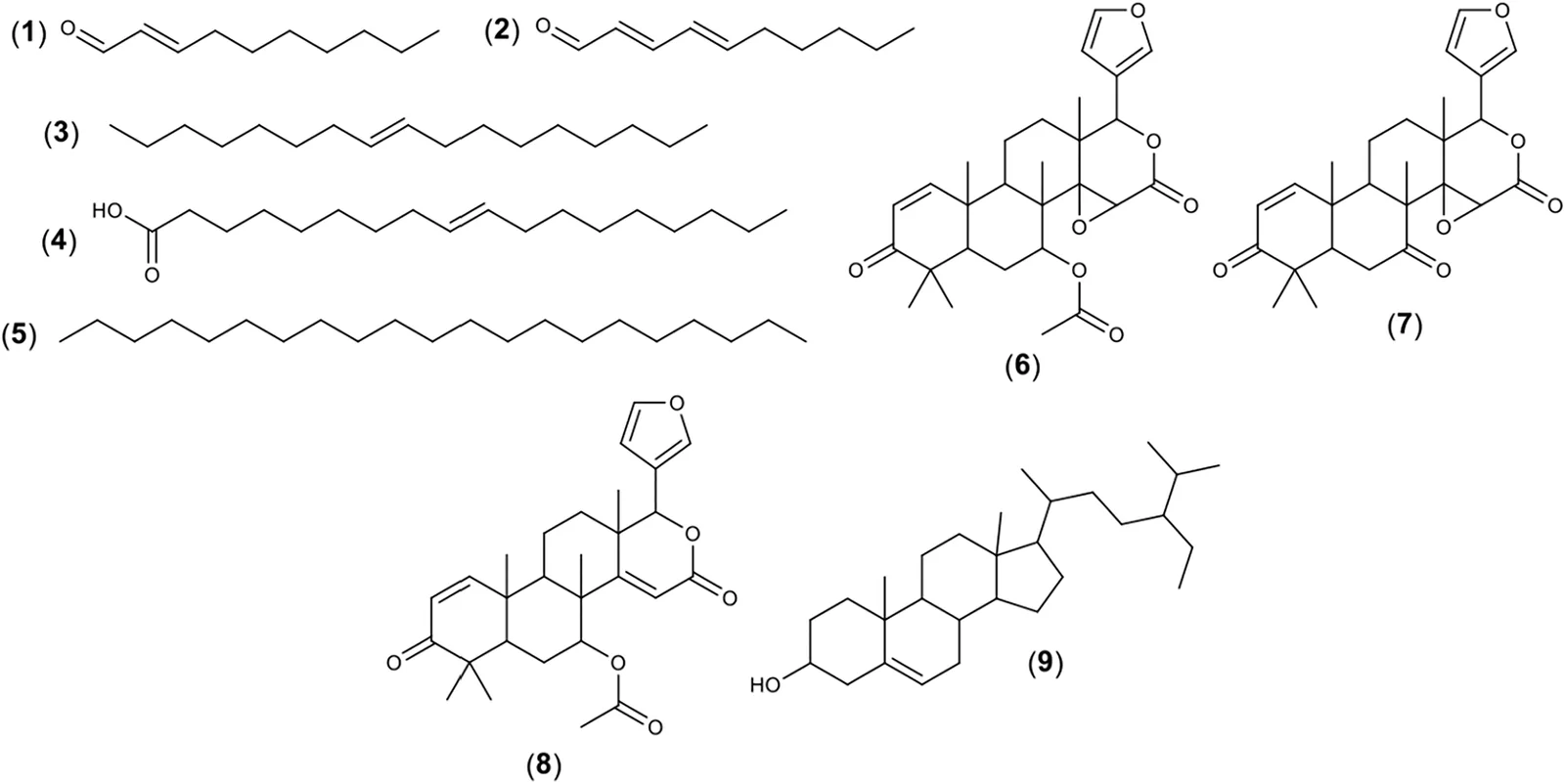
Chemical structure of compounds identified in andiroba oil by GC-MS: (1) 2E-Decenal; (2) 2,4-Decadienal; (3) 8-Heptadecene; (4) Oleic Acid; (5) Heneicosane; (6) Gedunin; (7) 7-Ketogedunin; (8) 14,15-Deoxogedunin; (9) β-Sitosterol.

**Table 1:** Chemical Characterization of Andiroba Oil

***N*°**	**compound identified**	**retention time (min)**	**relative abundance (%)**
1	2-decenal	6.496	4.231
2	2,4-decadienal	7.21	3.217
3	8-heptadecene	11.466	1.026
4	oleic acid	24.334	38.232
5	heneicosane	24.802	3.442
6	gedunin	30.695	22.671
7	7-ketogedunin	31.956	13.955
8	14,15-deoxogedunin	34.357	6.679
9	β-sitosterol	39.693	4.499

Gedunin, one of the main limonoids identified in andiroba oil (22.67%), is widely recognized as a key contributor to the oil’s biological activity.^14,15^ This tetranortriterpenoid displays a wide range of pharmacological properties, including anti-inflammatory, antioxidant, anticancer, antimicrobial, and insecticidal effects.^15^ In this study, gedunin (**6**, 22.67%) and its oxidized derivatives (7-ketogedunin (**7**, 13.95%) and 14,15-deoxogedunin (**8**, 6.67%)) represented a significant portion of the secondary metabolites detected by GC-MS, emphasizing their relative abundance and importance.

Chronic wounds, especially diabetic wounds, exhibit changes in the healing process characterized by persistent inflammation. In a study using a mouse acute arthritis model, gedunin reduced the levels of inflammatory markers IL-6, TNF-α, LTB_4_, and PGE_2_, exhibiting anti-inflammatory and pain-relieving effects, as well as a decrease in neutrophil infiltration at the inflammatory site.^16^ These results highlight the potential therapeutic use of formulations containing gedunin-rich andiroba oil for the treatment of chronic wounds.

ß-Sitosterol is a phytosterol with a structure similar to cholesterol. Studies demonstrate the potential of this compound as a pro-healing agent, presenting angiogenic effects and stimulating collagen deposition.^17^ A clinical trial involving children up to five years of age with burn wounds treated with an ointment containing β-sitosterol observed accelerated healing, including early formation of granulation tissue, rapid eschar liquefaction, and complete absorption of skin grafts.^18^

The high level of oleic acid (**4**, 38.23%) was expected given its known role in andiroba oil’s fatty acid profile. Long-chain alkene 8-heptadecene (**3**, 1.026%) and the hydrocarbon heneicosane (**5**, 3.442%), were also identified. Due to their low polarity and hydrophobic nature, these molecules may contribute to the internal lipid core of the nanodroplets, potentially helping to maintain the droplet structure and influencing viscosity.^19^ Biologically, oleic acid has anti-inflammatory effects, supports skin repair, and enhances transdermal delivery by increasing membrane permeability.^20^

The degradation of fatty acids leads to the formation of aldehydes such as 2-Decenal, and 2,4-Decadienal, which were found at low levels in andiroba oil (4.23 and 3.21%, respectively). These compounds are well-known byproducts of lipid peroxidation and are commonly used as markers of oxidative deterioration in oils and emulsions.^21^

### 3.2 Production of Andiroba Oil Gel-Based Nanoemulsion

Regarding the andiroba oil-based nanomulsion developed in previous work by our research group,^9^ the formulations developed in this work were modified with respect to the addition of 1 co-surfactant (PEG-40), an increase in the alternating pulse sonication time from 3 to 10 min, the addition of different concentrations of andiroba oil, and incorporation into a carbopol gel matrix. These changes optimized the physicochemical characteristics of the formulation, resulting in a reduction in the average nanodroplet size of the nanoemulsions from 205.7 nm (previous work^9^) to 131.04 nm (NeAnd and NeAndG), polydispersity index from 0.295 to 0.186 (NeAnd and NeAndG), and ζ potential from −4.16 mV to –29.93 mV (NeAnd) and –74.50 mV (NeAndG).

The present study was designed to compare the physicochemical characteristics, stability behavior, and initial in vitro biological profile of the liquid nanoemulsion (NeAnd) and its gel-based counterpart (NeAndG). We aimed to obtain a semisolid system that could be more suitable for future skin-related applications, since gel matrices may improve formulation handling and promote better retention at the site of administration. For wound application, hydrogel-based formulations are widely used because they offer good spreadability and a hydrophilic surface that creates a moist microenvironment, which is favorable to the healing process, especially in dry wounds.^22^ Carbopol gel is a water-soluble polymer commonly used in various topical formulations, mainly due to its high biocompatibility.^23^ Furthermore, the polymeric matrix of the gel reduces the movement of nanodroplets in the nanoemulsion, preventing coalescence and promoting the stability of the formulation.^22,23^

As shown in Table 2, there were no significant changes in the hydrodynamic diameter or polydispersity index after incorporation into the gel (NeAndG). Due to the anionic nature of the Carbopol gel, the association resulted in a significant reduction in the ζ potential, from -29.9 mV to -74.5 mV (average values for NeAnd and NeAndG, respectively).

**Table 2:** Hydrodynamic Diameter, PdI, ζ Potential, and pH of Andiroba Oil Nanoemulsion (NeAnd) and Its Gel Form (NeAndG)

**formulations** ^a^	**hydrodynamic diameter (nm)**	**polydispersity index**	ζ **potential**	**pH**
**NeAnd 10**	127.67 ± 1.10	0.1961 ± 0.0116	–30.08 ± 1.42	5
**NeAnd 20**	127.67 ± 1.00	0.1938 ± 0.0288	–30.77 ± 0.66	5
**NeAnd 40**	119.47 ± 0.90	0.1513 ± 0.0145	–28.94 ± 2.35	5
**NeAndG 5**	147.03 ± 1.95	0.2257 ± 0.0038	–76.84 ± 0.42	6
**NeAndG 10**	133.40 ± 1.91	0.1889 ± 0.0068	–74.95 ± 5.15	6
**NeAndG 20**	128.40 ± 1.30	0.1649 ± 0.0214	–71.72 ± 9.16	6

a See topic Section 2.2 for details.

### 3.3 Long-Term Thermal Stability Analysis of NeAnd and NeAndG

Stability tests are crucial for identifying optimal storage conditions and ensuring quality control of formulations. To assess stability, NeAnd and NeAndG formulations were stored for 120 days at 4, 25, and 37 °C.

The macroscopic appearance of NeAnd and NeAndG after production was slightly white and translucent (Figure 2). When stored at 4 and 25 °C, all formulations showed no macroscopic changes. However, at 37 °C, NeAnd developed a yellowish color, first observed on day 30 for NeAnd 40, day 60 for NeAnd 20, and day 90 for NeAnd 10. Over time, this yellow coloration intensified (Figure 2). These color changes likely relate to lipid peroxidation of phospholipids in the surfactant and fatty acids in andiroba oil. The incorporation of the formulations in a hydrogel matrix delayed lipid peroxidation. NeAndG exhibited a yellowish coloration only in the NeAndG 20 formulation stored at 37 °C starting from day 60. This change was less pronounced than in NeAnd, possibly due to the higher gel density, which reduces molecular mobility, lipid exposure to oxygen, and the chain reactions associated with lipid peroxidation.^24^ At 37 °C, NeAnd 40 on day 60 and NeAnd 20 on day 90 exhibited macroscopic signs of phase separation (Figure 2).

**Figure 2:**
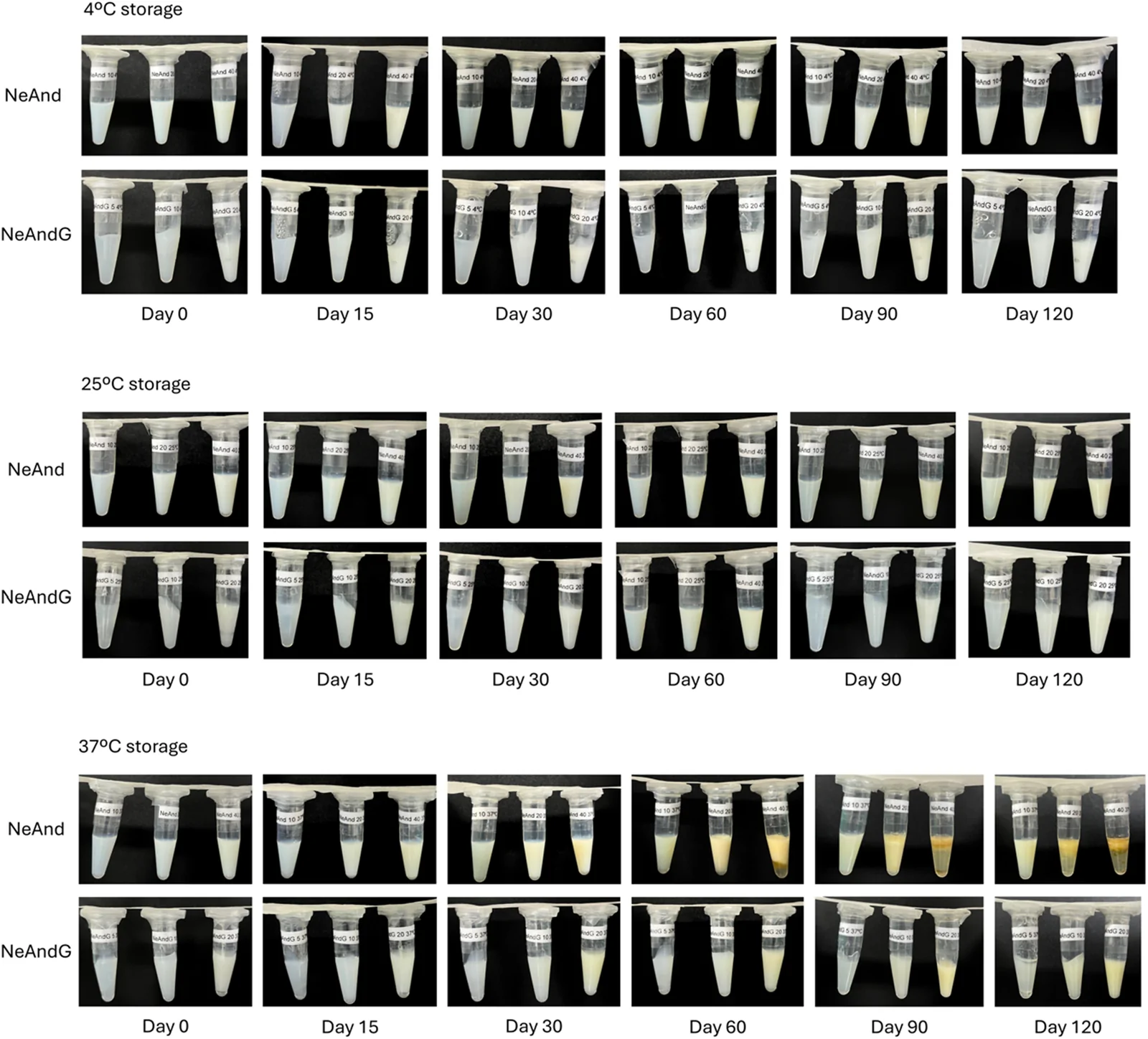
Effect of temperature and time of storage on the macroscopic aspect of andiroba oil nanoemulsion (NeAnd) and its gel form (NeAndG). Each image contains three eppendorf tubes in the following sequence: NeAnd 10, 20, and 40 and NeAndG 5, 10, and 20. Samples were stored at 4, 25, and 37 °C for 120 days under light protection, and the pictures were registered on days 0, 15, 30, 60, 90 and 120.

The reduction in pH of NeAnd 10, 20, and 40 to pH 3 after 120 days at 37 °C supports the possible formation of acidic molecules through lipid peroxidation, which was not observed in NeAndG, as it maintained its initial pH at 37 °C (Supporting Figure 1). Heat accelerates molecular reactions, facilitating interactions between oxygen and unsaturated fatty acids, resulting in free radicals that initiate lipid peroxidation.^25^ No color or pH changes were observed in NeAnd and NeAndG stored at 4 and 25 °C, suggesting that lipid peroxidation did not occur within 120 days of storage.

At temperatures of 4 and 25 °C, no significant changes in the DLS characterization were observed in NeAnd and NeAndG. The hydrodynamic diameter remained below 200 nm, and the polydispersity index below 0.3 (Figure 3). However, the macroscopic changes observed in the NeAnd formulations at 37 °C correlate with changes in the DLS data. Significant increases in hydrodynamic diameter and polydispersity index were observed in NeAnd 20 and 40 stored at 37 °C, with patterns directly proportional to the oil concentration and storage duration. After 120 days at 37 °C, NeAnd 20 and 40 showed increases of 581.16 nm and 1073.36 nm, respectively, compared to day 0 (*p* < 0.0001). In both formulations, after 120 days at 37 °C, the polydispersity index approached 1, indicating nanodroplet aggregation and phase separation. The macroscopic stability of gel formulations was consistent with the DLS data. Throughout the 120-day period, no significant changes in the hydrodynamic diameter, polydispersity index, or ζ potential of NeAndG were observed at any storage temperature (Figure 3).

**Figure 3:**
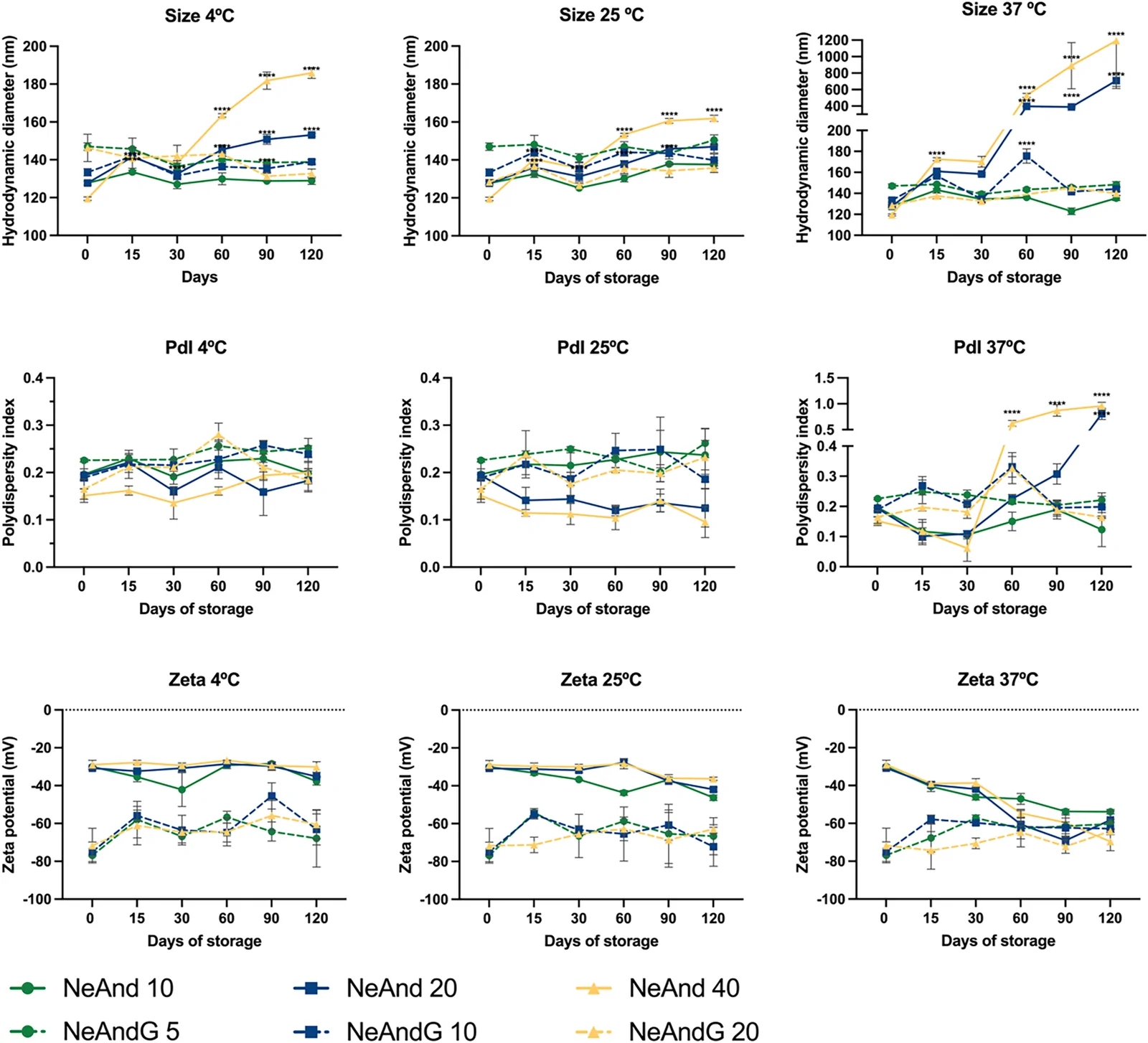
Effect of time and temperature on the hydrodynamic diameter, polydispersity index, and ζ potential of andiroba-based nanoemulsion (NeAnd) and its gel form (NeAndG). The data were subjected to normality analysis, and data with a normal distribution were analyzed using the paired one-way ANOVA test, while nonparametric data were analyzed using the paired Friedman test. Significant differences between each formulation and its day 0 at *p* < 0.0001 are represented with ****.

The influence of temperature on the physical stability of lipid nanostructures observed in this study corroborates the recent findings of Bezerra et al., who demonstrated that nanoemulsions based on copaiba balsam oil (*Copaifera sp.*) maintain superior structural integrity when stored under refrigeration (4 °C) compared to higher temperatures (25 and 37 °C).^26^

Applying centrifugal force to nanoemulsions serves as an important accelerated stability test because it promotes collisions between nanodroplets, increasing the possibility of coalescence with phase separation and creaming.^27^ NeAnd 10, 20, and 40 and NeAndG 5, 10, and 20 underwent centrifugation, and no macroscopic signs of instability or significant changes in the DLS characterization were observed (Supporting Table 1).

Variations in the pH of nanoemulsions directly affect the surface charge of the nanodroplets. The phospholipids in lecithin have ionizable groups in their structure, such as phosphate (−PO_4_^2–^), amino (−NH_3_^+^), and carboxyl (−COOH) groups.^28^ These groups tend to protonate and deprotonate depending on the pH of the surrounding medium, and these charge exchanges lead to changes in ζ potential.^29^ As expected, subjecting NeAnd 10 to pH levels of 3.0, 5.0, 7.0, 9.0, and 11.0 caused inverse changes between pH value and ζ potential, which ranged from 6.4 mV at the most acidic pH (3.0) to –66.8 mV at the most basic pH (11.0) (Figure 4) (*p* < 0.0001). Similarly, significant changes were observed in NeAndG 5, which had values of –4 mV at pH 3 and –66.1 mV at pH 11 (*p* < 0.0001).

**Figure 4:**
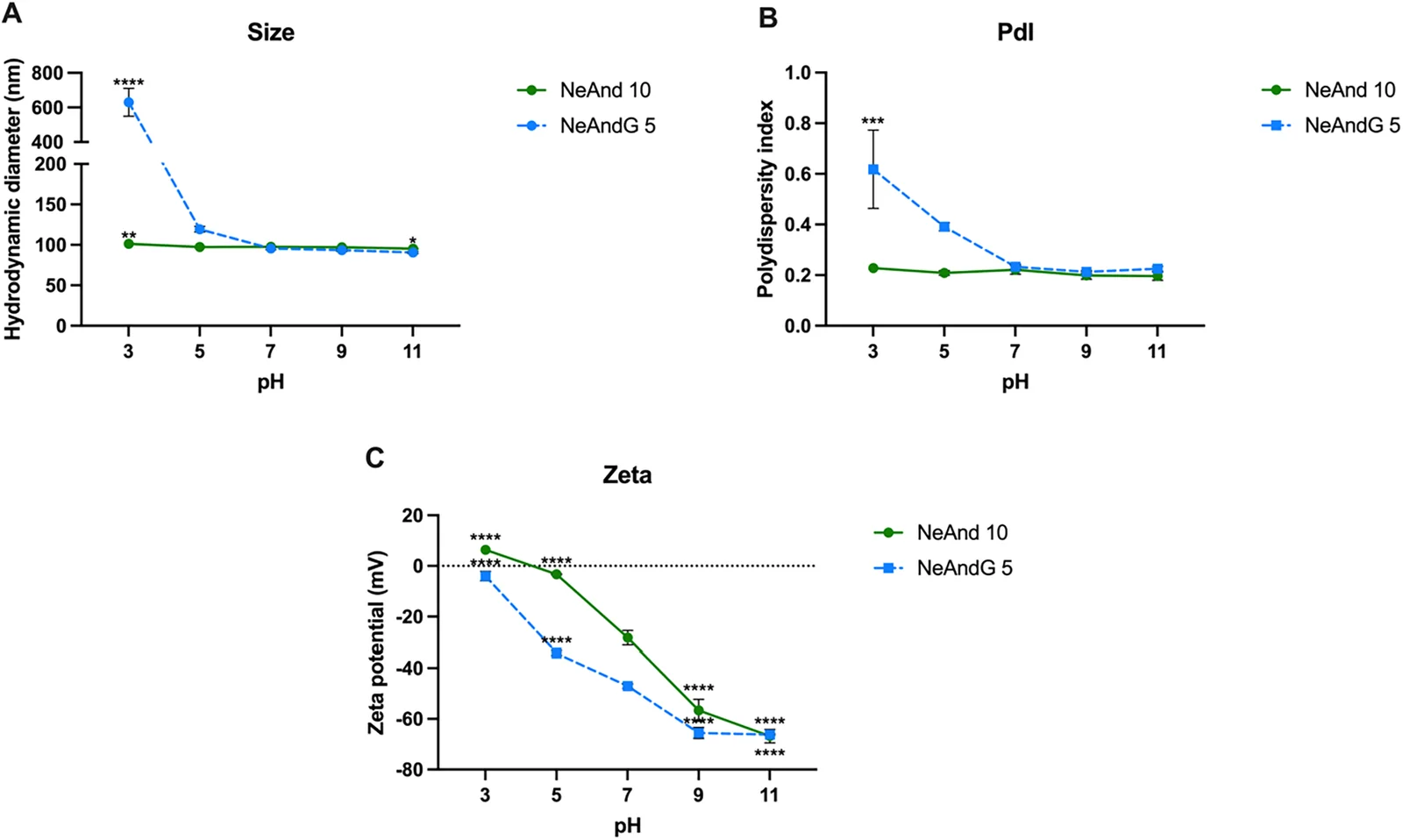
Effect of pH on the stability of andiroba-based nanoemulsion (NeAnd) and its gel form (NeAndG). NeAnd 10 and NeAndG 5 was evaluated through hydrodynamic diameter (A), polydispersity index (PdI) (B), and ζ potential (C). Values are expressed as mean ± SD. Statistical analysis was performed using one-way ANOVA (Tukey post hoc test). Significant differences between each formulation at different pH levels compared to pH 7 are indicated with * *p* < 0.05, ** *p* < 0.01, *** *p* < 0.001, and **** *p* < 0.0001.

Although the ζ potential of NeAnd 10 at pH 3 and 5 was near the isoelectric point (6.40 and -3.28 mV, respectively), there were no significant changes in the hydrodynamic diameter or polydispersity index compared to pH 7. However, in NeAndG 5 at pH 3, the ζ potential was close to the isoelectric point (-4.03 mV), and the hydrodynamic diameter increased from 95.57 nm (pH 7) to 629.86 nm (pH 3). The polydispersity index also increased from 0.232 (pH 7) to 0.618 (pH 3) (*p* < 0.05), indicating coalescence. The gelation agent used for NeAndG was Carbopol 940, which is composed of polyacrylic acid. The carboxyl groups (−COOH) in this acid are protonated at acidic pH, preventing electrostatic repulsion among the polymer chains and causing them to collapse.^30^ This gel destructuring may have contributed to the instability of the nanodroplets. However, it is important to note that the DLS and ELS analyses were performed on diluted gel samples with a liquid-like character, which limits the understanding of the nanogel’s properties in its semi-solid state.

In contrast, at basic pHs (9 and 11), both NeAnd and NeAndG exhibited a significant increase in ζ potential modulus compared to neutral pH (7) (*p* < 0.0001). For NeAnd, this increase was from 28.3 mV at pH 7 to 66.8 mV at pH 11, and for NeAndG, it was from 47.0 mV at pH 7 to 66.1 mV at pH 11. This increase in surface charge prevented coalescence and enhanced the stability of the nanodroplets, which remained stable in terms of hydrodynamic diameter and polydispersity index in both formulations (Figure 4).

It is important to highlight that the storage, pH, and thermal stability conclusions reported here are based on physicochemical and macroscopic parameters rather than on post-storage quantification of secondary metabolites. The preservation of key bioactive constituents after storage and oxidative degradation studies, should be considered in future investigations focused on the chemical stability of the formulation.

### 3.4 Fourier-Transform Infrared Spectroscopy (FTIR) Characterization of NeAnd and NeAndG

The FTIR spectra of the isolated surfactants, oily phase (andiroba oil), and NeAnd are shown in Figure 5. Supporting Table 2 provides the assignments of vibrational modes and the corresponding wavelength numbers (cm^–1^) for each sample.

**Figure 5:**
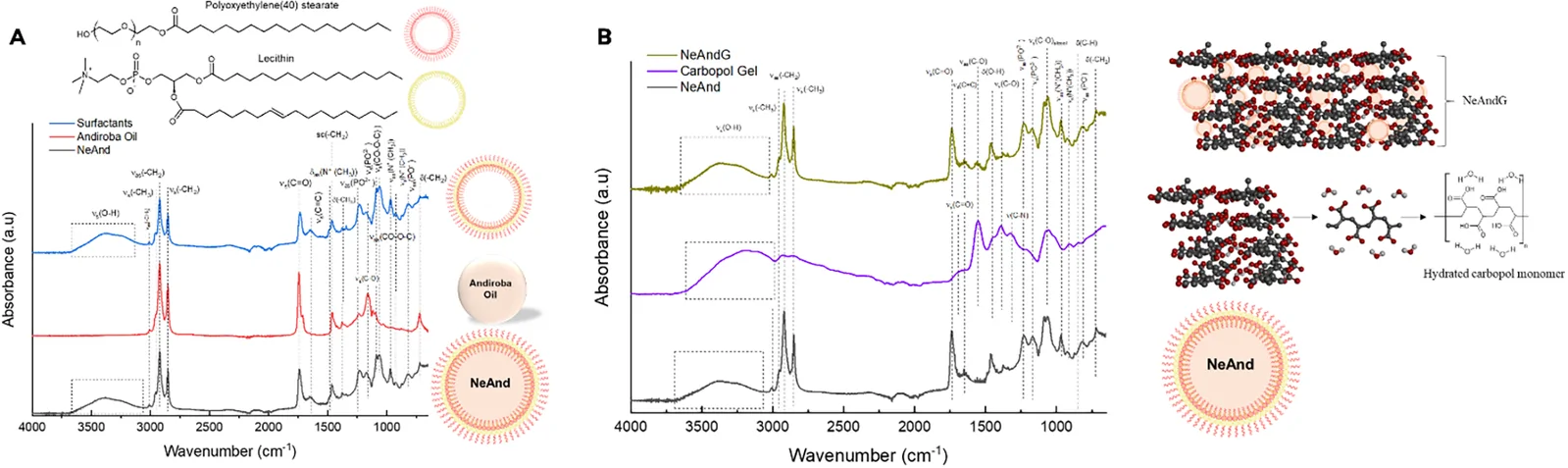
FTIR spectra of (A) surfactants (Lecithin and PEG 40), andiroba oil, and andiroba-based nanoemulsion (NeAnd) (B) mixture of andiroba-based nanoemulsion (NeAnd), pure Carbopol gel base, and Carbopol gel base containing NeAnd (NeAndG) and schematic representation of samples structures.

In the specific case of surfactants and NeAnd, a broad band is observed between 3210 and 3060 cm^–1^, which originates from the stretching of the O–H bond present in polyoxyethylene (40) stearate. The characteristic groups of lecithin are detected in the spectrum of surfactants and nanoemulsions (NeAnd): deformation vibration of the N^+^(CH_3_) group, asymmetric and symmetric stretching vibrations of the –PO_2_^–^ group, asymmetric stretching vibration of the PO^–^ bond, and symmetric and asymmetric stretching vibrations of CO–O-C bonds in the region from 1520 to 800 cm^–1^ in surfactants originating from the surfactant and co-surfactant. C–H bending vibrations are identified between 1386 and 1321 cm^–1^. In the nanoemulsion (NeAnd), there are more chemical groups from the surfactants than from the oil phase due to their larger mass proportion.

A structural evaluation of the NeAndG compared to the NeAnd and Carbopol gel sample using FTIR spectroscopy is shown in Figure 5, with vibrational mode assignments listed in Supporting Table 2. The Carbopol gel base structure is characterized by a broad O–H stretching vibration band between 3621 and 2990 cm^–1^. This band remains in the NeAndG sample, while the symmetric stretching of −CH_3_ and the asymmetric as well as symmetric stretching of −CH_3_ are less intense in the Carbopol gel base but become more pronounced after incorporating NeAnd. The hydration process of Carbopol to form the hydrogel causes the displacement of the symmetric stretch band of the C=O bond to lower wavenumbers, as detailed in Supporting Table 3. However, in the NeAndG nanogel, the band shifts to higher wavenumbers, indicating changes in the hydration process.

The stretching vibration of the C–N bond can be explained by the triethanolamine used to raise the pH to form the hydrogel. The NeAnd vibration bands shown in Figure 5 and Supporting Tables 2 and 3, previously discussed, indicate the presence of vibrational modes of the surfactants, mainly the lecithin groups, which confirms the incorporation of the nanodroplets into the hydrogel structure.

### 3.5 Thermal Analysis

In Figure 6, TGA and DTA data for the NeAnd, pure Carbopol Gel Base, and NeAndG are presented. For the NeAnd, a slight decrease in mass is observed due to the loss of water weakly bound to the structure up to a temperature of 250 °C, along with a structural modification marked by a phase change that makes the sample more fluid. This process is confirmed by the complex profile of the DTA curve in this temperature range. From 300 °C onward, an abrupt loss of mass begins, indicated by an endothermic process on the DTA curve. This behavior aligns with data in the literature,^6,31^ which indicates that andiroba oil degradation starts around 300 °C. The second endothermic event, accompanied by continuous mass loss between 400 and 600 °C, may be related to the degradation of surfactants. The subsequent events suggest complete degradation of the sample with the release of small amounts of energy.

**Figure 6:**
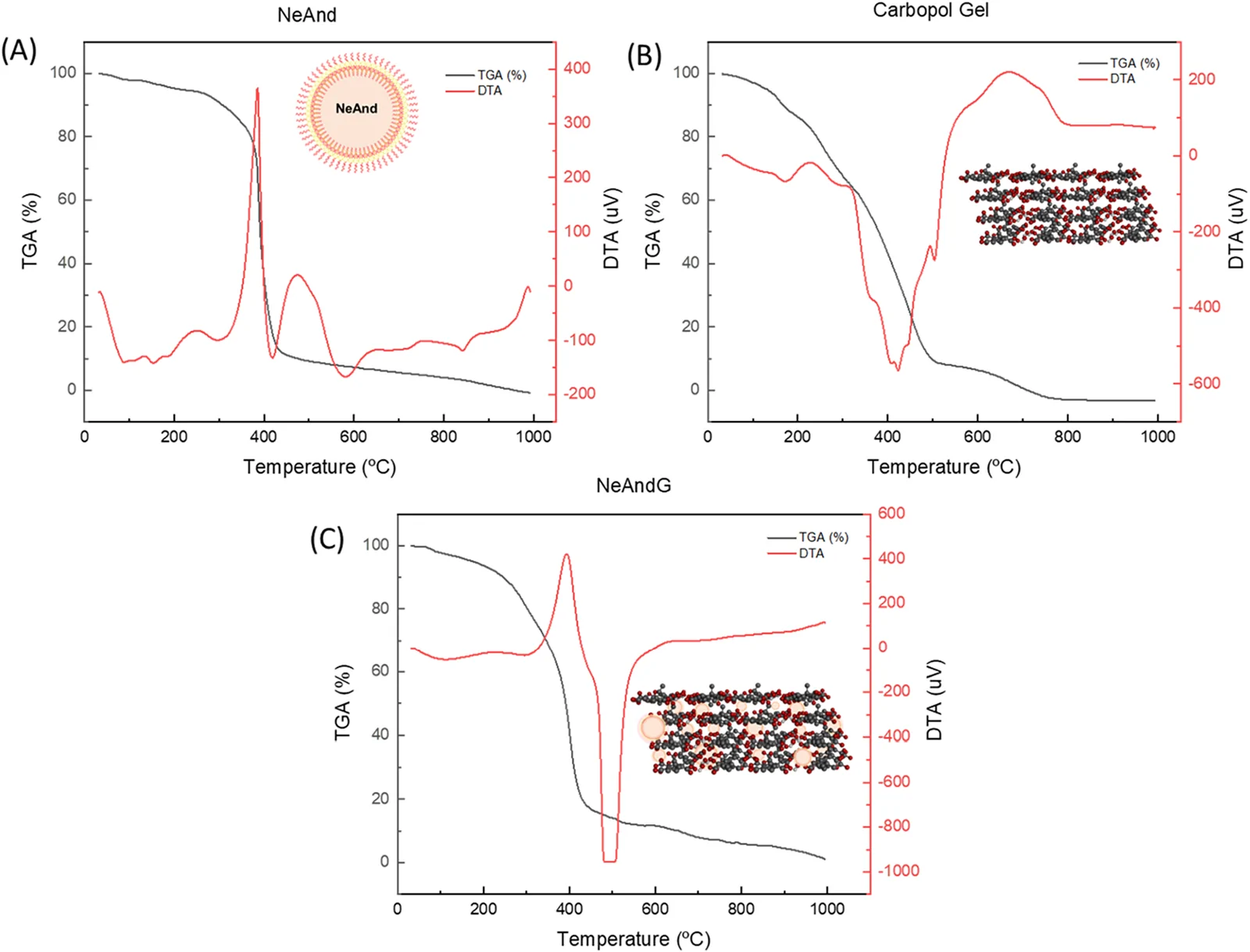
TGA and DTA curves of andiroba-based nanoemulsion (NeAnd) (A), pure Carbopol gel base (B), and Carbopol gel base containing NeAnd (NeAndG) (C).

For the pure Carbopol gel base, water loss occurs at 50 °C, indicating that most of the hydrogel’s mass is water molecules. Between 200 and 300 °C, energy absorption shown by the DTA curve likely corresponds to melting and structural changes in the hydrogel. Starting at 300 °C, a sharp mass loss occurs alongside an exothermic energy flow, which may relate to the decarboxylation process.^30^ Finally, above 550 °C, a more gradual mass loss, accompanied by an endothermic process, likely corresponds to evaporation of residual substances.

The TGA and DTA curves for the NeAndG sample show a change in the hydrogel’s thermal behavior due to NeAnd presence. Water loss with structural changes occurs up to 250 °C, accompanied by energy absorption. Between 340 and 430 °C, there is an abrupt mass loss during an endothermic process, similar to what was observed for NeAnd, followed by exothermic processes at 440 and 630 °C, which resemble the behavior of the Carbopol gel base alone. This modification can be explained by the formation of new chemical interactions, as indicated in the FTIR spectra. Compared to the Carbopol gel base, NeAndG contains fewer water molecules, suggesting that nanodroplets occupy some spaces normally filled by water. However, the hydrogel’s properties are not significantly affected, as the polar groups of the surfactants interact with the polar groups of Carbopol molecules.

### 3.6 Cell Viability

After 24 h of exposure to NeAnd and NeAndG at concentrations of 62.5 to 2000 μg/mL, keratinocyte (HaCaT) cell viability was reduced in a dose-dependent manner (Figure 7A). Cell viability remained above 80% at concentrations up to 250 μg/mL across all tested treatments. Starting at 500 μg/mL, a significant decrease compared to the negative control (DMEM) was observed in the NeAnd, OL, and OLG treatments, which showed viability rates of 67.7, 71.2, and 73.9%, respectively (*p* < 0.05). For NeAndG, a significant viability reduction was seen from 1000 μg/mL onward, with 66.2% viability. The lowest cell viability was at the highest concentration of 2000 μg/mL, with NeAnd, NeAndG, OL, and OLG groups viability percentages of 47.7, 52.8, 39.7, and 36.1%, respectively (*p* < 0.0001). Comparing viability at 2000 μg/mL among these groups, OL and OLG treatments caused a greater reduction in viability compared to NeAnd and NeAndG (*p* < 0.05).

**Figure 7:**
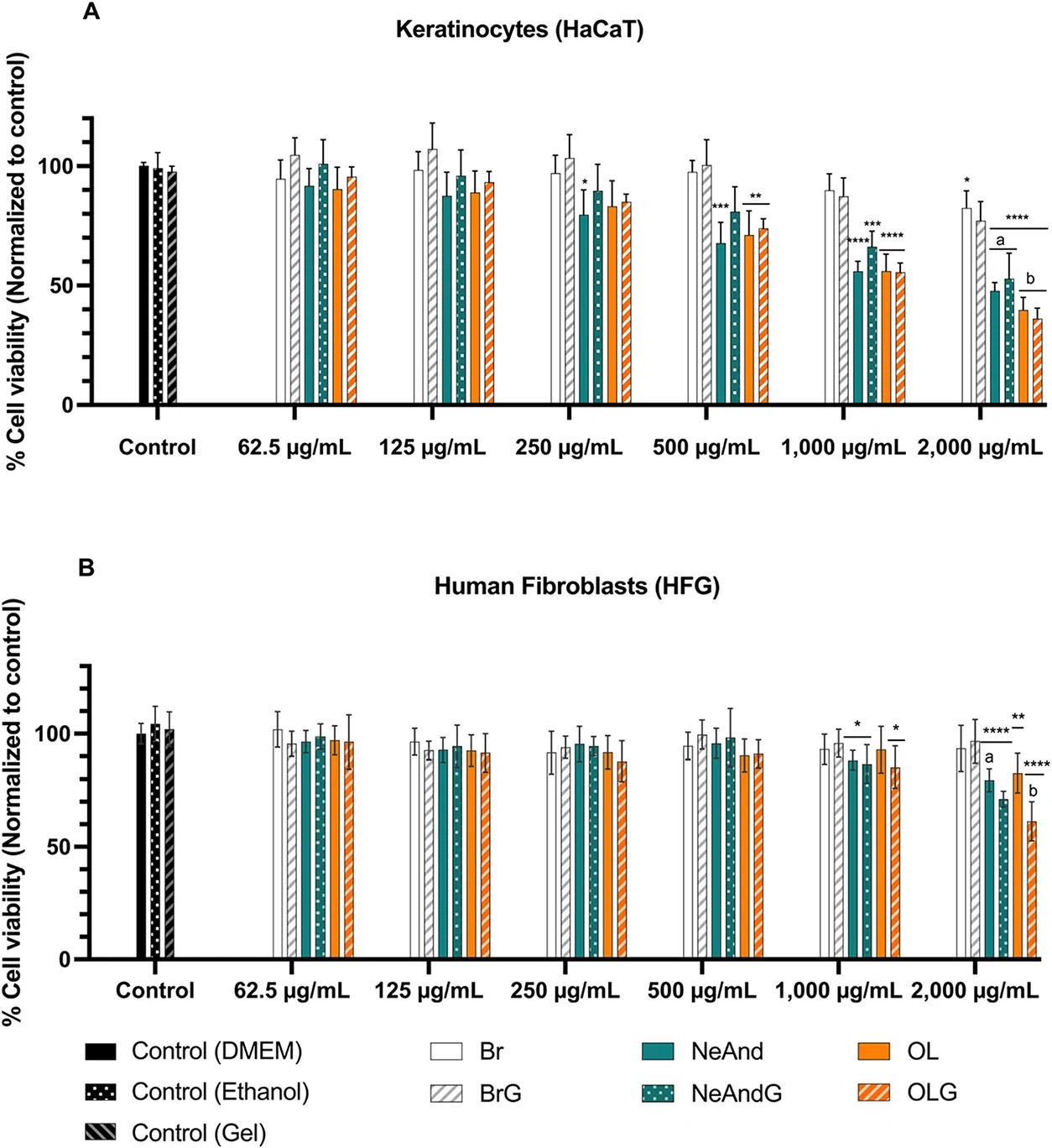
Viability of keratinocytes (HaCaT) (A) and human fibroblasts (HGF) (B) exposed for 24 h to andiroba-based nanoemulsion (NeAnd) and its gel form (NeAndG), Carbopol (gel), free andiroba oil (OL) and its Gel form (OLG), blank (Br) and gel form (BrG). Viability results were expressed as mean and standard deviation. Statistical analysis was conducted using Kruskal-Wallis test. Significant differences between each group and control (DMEM) are represented with * *p* < 0.05, ** *p* < 0.01, *** *p* < 0.001, **** *p* < 0.0001. Significant differences between NeAnd, NeAndG, OL, and OLG are represented with letters a, b.

Human fibroblasts (HGF) demonstrated greater resistance to the effects of treatments compared to keratinocytes. In fibroblasts, all treatments maintained cell viability above 80% up to a concentration of 1000 μg/mL (Figure 7B). At 2000 μg/mL, differences compared to the negative control group (DMEM) appeared in the NeAnd, NeAndG, OL, and OLG groups, which showed viability of 79.5, 71.2, 82.6, and 61.3%, respectively (*p* < 0.0001). When comparing these four groups at 2000 μg/mL, a significant difference was found between NeAnd and OLG, with OLG decreasing viability more substantially than NeAnd (*p* < 0.05).

One of the main challenges in developing nanoformulations is choosing non-cytotoxic excipients. In particular, surfactants, because of their amphiphilic nature, tend to interact with the lipid bilayer, leading to structural disorganization, increased fluidity, pore formation, and cell lysis at high concentrations.^32^ Interestingly, in both fibroblasts and keratinocytes, groups containing only surfactants (Br and BrG), Carbopol gel, and ethanol control did not show a significant reduction in viability at any tested concentrations, indicating that the formulation excipients do not affect cell viability.

The differences in the cytotoxicity of the free oil compared to the nanostructured oil are similar to those observed by Milhomem-Paixão et al., in which free andiroba oil showed lower IC50 values in murine fibroblasts (NIH3T3) compared to the nanostructured oil, 0.5353 and 1.191 mg/mL, respectively. In the same study, morphological analysis of the cells demonstrated changes in cellular morphology that demonstrate the possible effect of the oil on the cell membrane.^33^ The attenuation of the cytotoxic effect of the nanostructured oil compared to the free oil demonstrates the potential of nanostructuring to circumvent the cytotoxicity of the free oil, optimizing cellular interaction through different cellular internalization pathways.

### 3.7 Scratch Wound Healing Assay of NeAnd and NeAndG

The migration of fibroblasts and keratinocytes was evaluated using the scratch assay to assess the wound-healing potential of the tested formulations. The results, shown as the percentage of wound closure, are presented in Figure 8.

**Figure 8:**
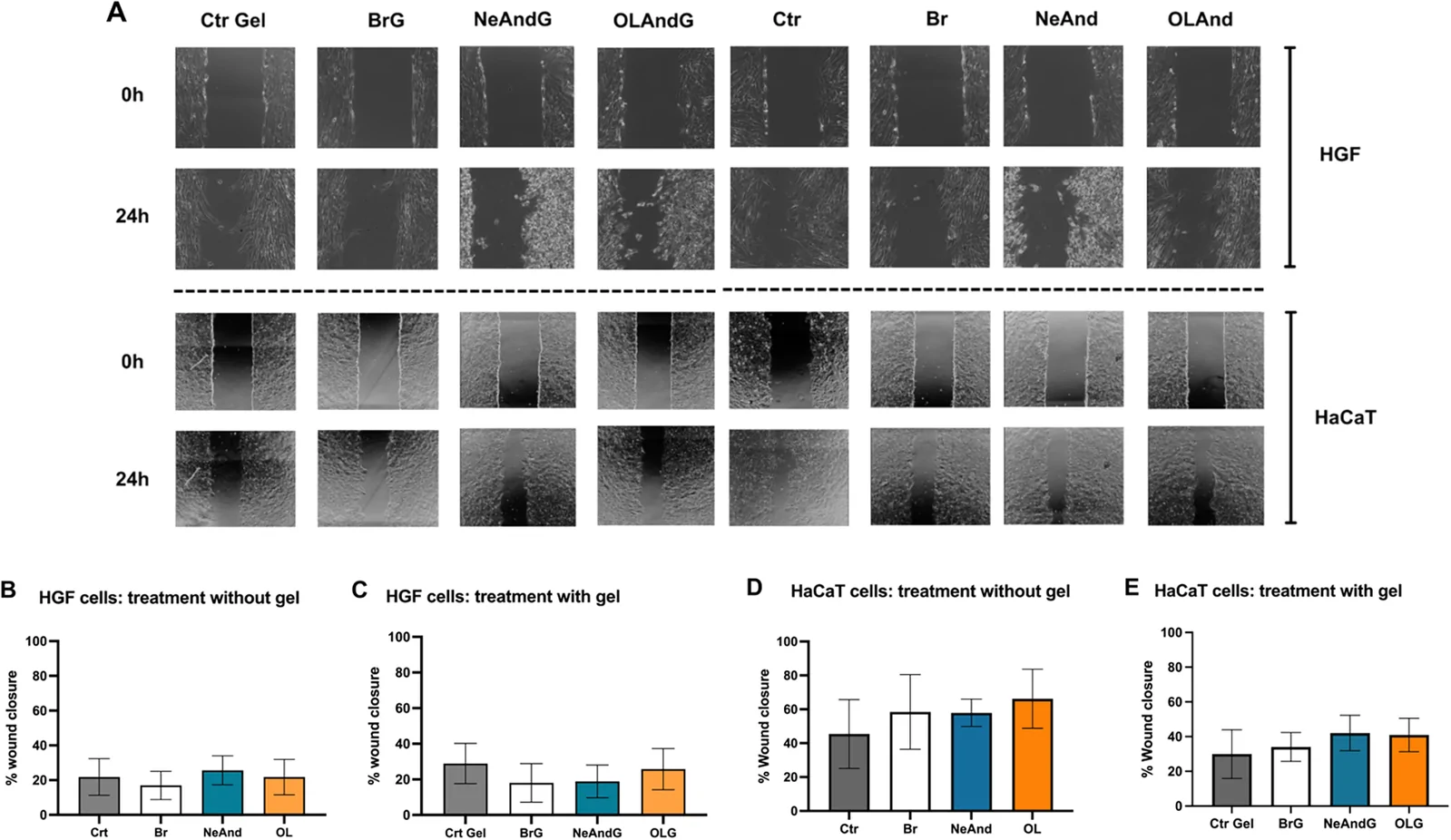
Effect of different treatments on wound closure in HGF and HaCaT cells, with and without gel formulation. (A) Representative images of HGF and HaCaT cell migration before (0h) and after (24h) incubation with treatments. Bar graphs show the percentage of wound closure in HGF fibroblasts and HaCaT keratinocyte monolayers treated for 24 h: (B) HGF without gel; (C) HGF with gel; (D) HaCaT without gel; (E) HaCaT with gel. Treatments include untreated control (Ctr/CtrG), nanoparticles without oil (Br/BrG), nanoemulsion containing andiroba oil (NeAnd/NeAndG), and pure andiroba oil (OL/OLG). The suffix “G” indicates formulations with gel. The treatments concentrations were 250 μg/mL for HaCat and 1000 μg/mL for HGF. Images were captured using a 10 × objective lens. Data are shown as mean ± standard deviation (SD), with error bars representing variability across replicates.

In keratinocytes, Figure 8A,D show the treatments without gel, where all experimental groups (Br, NeAnd, and OL) exhibited wound closure comparable to the control group (Ctr), with all values ranging from 45 to 66%. Although the treatment with andiroba oil (OL) showed the highest percentage of closure (66.2%), it was not possible to observe statistically significant differences among the groups. The incorporation of the treatments into a gel (Figure 8A,E) did not have a significant effect on keratinocyte migration.

Similarly, in fibroblasts, as shown in Figure 8A,B, which depict the treatment of cells without gel incorporation, all experimental groups (NeAnd, Br, and OL) exhibited wound closure below 30%, similar to the control group (Ctr). Although the group treated with NeAnd showed the highest closure percentage (25.6%), no statistically significant differences were found among the groups. Incorporating the treatments into a gel did not produce significant differences between the groups.

According to Jacob and Nair (2026), converting nanoemulsions into nanogels protects lipophilic actives from oxidative degradation and volatilization while mimicking the moist extracellular environment. For andiroba oil, this hybrid framework preserves the chemical integrity of bioactive constituents and prolongs residence time and occlusion at the wound site, which may be relevant for future topical formulation strategies.^34^ However, a key limitation in evaluating the in vitro biological performance of hydrogels using cell culture models lies in the requirement to dilute the formulation in liquid culture media. This dilution disrupts the polymeric network of the gel, leading to the release of nanoemulsion droplets and consequently eliminating the structural integrity of the nanogel system. As a result, the physicochemical distinctions between the nanogel and the nanoemulsion are no longer preserved under these conditions.

The scratch assay is a semi-quantitative model that assesses cell migration. Variations in some experimental factors tend to result in high standard deviation values, which directly impact statistical analysis and limit the assessment of treatment effects.^35–37^ In our previous work, a pro-migratory effect of NeAnd was observed in keratinocytes.^9^ Nevertheless, the scratch assay methodology used did not take into account the total area of the whole scratch. In the present study, this methodology was improved, and the total area of each wound in the wells was considered. The lack of statistical differences among the groups indicates that, under the tested experimental conditions, the treatments did not significantly accelerate the migration of fibroblasts or keratinocytes compared to their respective controls. Despite the limitations of the scratch assay, the results indicate that the healing effect of andiroba oil observed in more complex *in vivo* models described in the literature may involve other mechanisms of healing not directly related to cell migration. Analyses that assess other aspects of the healing process, such as cell proliferation, collagen production, and inflammation-related markers, are necessary to complement the migration test and provide more comprehensive data on the in vitro biological activity of formulations containing andiroba oil.

Furthermore, in vivo cutaneous wound models provide a more comprehensive assessment of healing outcomes, as they capture the complex and dynamic interactions involved in the entire repair process. The critical relevance of in vivo models for the validation of biomaterials is highlighted by the findings of Basu et al.. Although their nanofibrillated cellulose hydrogels showed no significant difference compared to the control in the in vitro scratch assay, it was revealed an opposite and superior outcome in a in vivo wound model. In vivo, the system overcame the limitations of cell culture by acting as a functional extracellular matrix, promoting accelerated macroscopic wound closure, intense angiogenesis, and the formation of a perfectly structured and stratified neoepithelium.^38^ In this context, our research group is currently investigating the effects of NeAndG on wound healing in diabetic animal models to further explore this issue.

## 4 Conclusion

The use of products derived from Brazilian biodiversity in skin-related applications has become increasingly popular. Scientific validation of these products is important not only to ensure their efficacy and safety but also to add value to these compounds. The incorporation of andiroba oil into a gel-formulated nanoemulsion represents a relevant advance in the development of nature-derived nanostructured systems. In the present study, the chemical characterization of andiroba oil revealed a composition rich in limonoids, which may contribute to its reported healing properties. Incorporating the oil into a nanoemulsion system, followed by its association with a gel matrix, resulted in a semisolid system that maintained the ideal characteristics of a nanoemulsion (hydrodynamic diameter <200 nm, polydispersity index <0.3, and negative surface charge) and exhibited improved thermal stability compared to formulations without the gel matrix. In this study, the nanoemulsion and nanogel interacted with key cells involved in healing (keratinocytes and fibroblasts) and showed a dose-dependent decrease in cell viability. The scratch assay results did not show an increased effect of the formulations on the migration of keratinocytes and fibroblasts. The present findings support the characterization and initial in vitro evaluation of these formulations in wound healing-related cells. Additional studies addressing other biological mechanisms, such as proliferation, collagen production, inflammation-related pathways, and in vivo repair models, are necessary to better define the relevance of these systems for future therapeutic investigation. Overall, this study integrates nanotechnology, traditional medicine, and regenerative biology, providing a physicochemically stable and biologically relevant nanostructured platform for further preclinical investigations.

## Supplementary Material



## Notes

Hydrodynamic diameter, polydispersity index, and ζ potential before and after centrifugation (Table S1); pH stability as a function of time and temperature (Figure S1); FTIR wavenumber assignments of surfactants, andiroba oil, and nanoemulsions (Table S2); and FTIR spectral comparison of nanoemulsion, gel base, and nanoemulsion-loaded gel (Table S3) (PDF)
