# Supplementary material for: Andiroba Oil (Carapa guianensis Aublet) Gel-Based Nanoemulsion: Development, Characterization, and In Vitro Evaluation in Wound Healing-Related Cells
Source: ACS Omega. 2026 Oct 1;11(40):60734–47. doi: 10.1021/acsomega.6c02635 (PMC13670253; doi:10.1021/acsomega.6c02635)
Supplement: Supplementary file 1 [file ao6c02635_si_001.pdf]

Andiroba Oil (*Carapa guianensis* Aublet) Gel-based Nanoemulsion: Development, Characterization, and *in vitro* Evaluation in Wound Healing-Related Cells

Vitória R. P. Silva[a,b], Atailson O. da Silva[c], Mosar C. Rodrigues[a,f], Karen R. Py-Daniel[a], Bruna R. B. Gomes[d]; Elysa B. O. Damas[a], Thalya S. R. Nogueira[e], Ivo J. C. Vieira[e], Mariana S. Castro[d]; Marcelo H. Sousa[c], Marcella L. B. Carneiro[a,f], Graziella A. Joanitti [a,b,f *]

[a]*Laboratory of Bioactive Compounds and Nanobiotechnology (LBCNano), University of Brasilia (UnB), Campus Darcy Ribeiro, Brasília, DF 70910-900, Brazil.*

[b]*Post-Graduation Program in Pharmaceutical Science, University of Brasilia (UnB), Campus Darcy Ribeiro, Brasília, DF 70910-900, Brazil.*

[c]*Green Nanotechnology Group, University of Brasilia (UnB), Centro Metropolitano, Ceilandia Sul, Brasília, DF 72220-275, Brazil.*

[d]*Laboratory of Protein Chemistry and Biochemistry, Department of Cell Biology, Institute of Biological Sciences, University of Brasilia, Brasilia, DF 70910-900, Brazil*

[e]*Laboratory of Chemical Sciences (LCQUI), State University of Northern Fluminense Darcy Ribeiro (UENF), Avenida Alberto Lamego 2000, Campos dos Goytacazes, RJ 28013-602, Brazil*

[f] *Post-Graduation Program in Nanoscience and Nanobiotechnology, University of Brasilia (UnB), Campus Darcy Ribeiro, Brasília, DF 70910-900, Brazil.*

*Corresponding author at: Laboratory of Bioactive Compounds and Nanobiotechnology (LBCNano), University of Brasilia (UnB), Campus Darcy Ribeiro, Brasília, DF 70910-900, Brazil.

Email address: gjoanitti@unb.br

Supporting Information

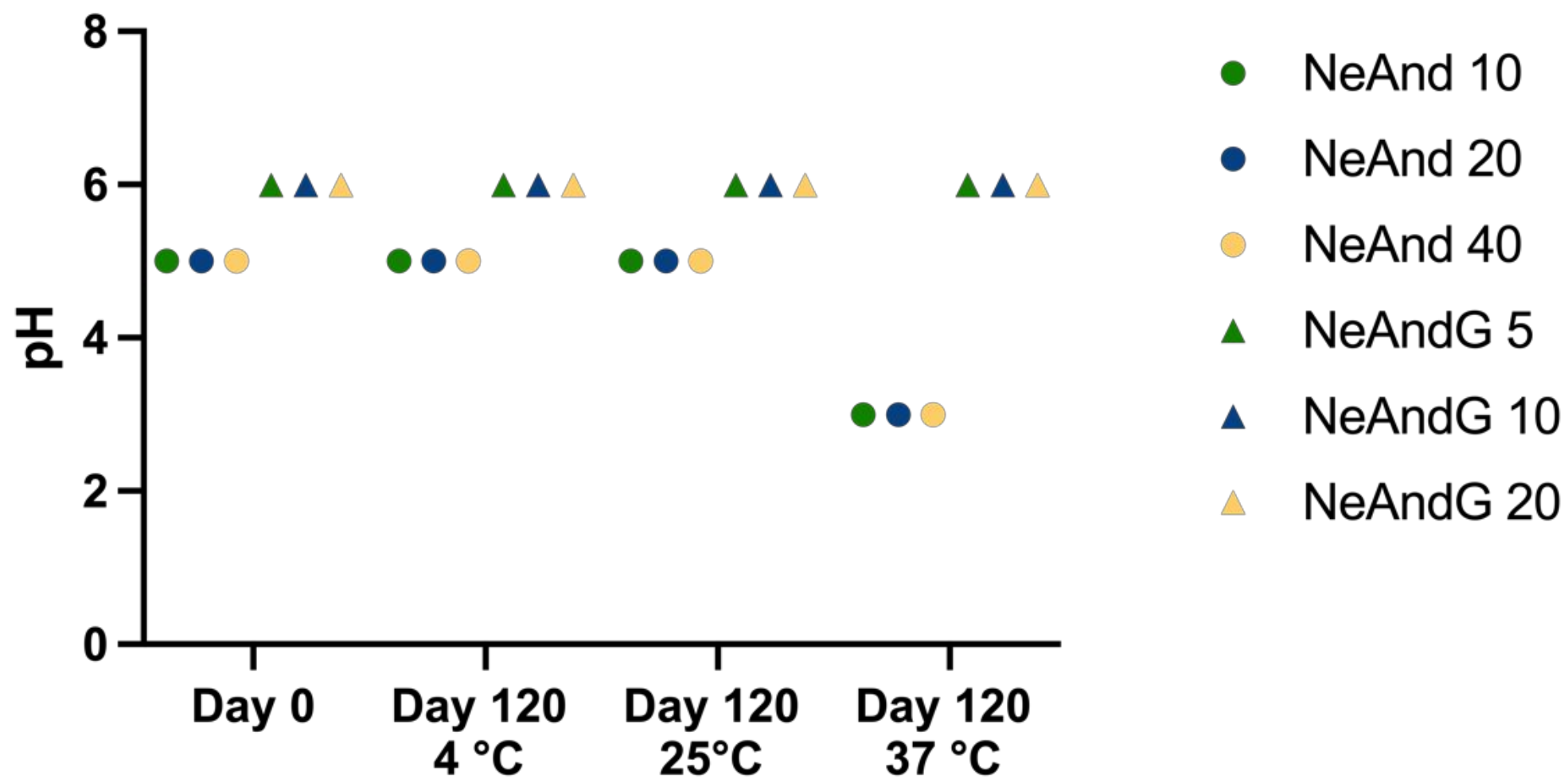

Supplementary Figure 1. Effect of time and temperature on pH of andiroba oil nanoemulsion (NeAnd) and its gel form (NeAndG). NeAnd 10, 20, and 40 correspond to nanoemulsion containing 10, 20, and 40 mg/mL of andiroba oil, respectively; NeAndG 5, 10, and 20 correspond to nanoemulsion incorporated into

a gel matrix with a final concentration of andiroba oil of 5, 10, and 20 mg/mL respectively. Statistical analysis was performed using Friedman test. No significant differences were observed.

Supplementary table 1. Hydrodynamic diameter, polydispersity index and zeta potential of andiroba-based nanoemulsion (NeAnd) and its gel form (NeAndG) before and after centrifugation at 9.500 RCF for 30 min at 25 °C.

Formulations	Hydrodinamic diameter (nm)		Polydispersivity index		Zeta potential (mV)	
	Before	After	Before	After	Before	After
**NeAnd 10**	127.9 ± 1.4	148.0 ± 0.5	0.172 ± 0.021	0.212 ± 0.013	-27.79 ± 1.03	-29.87 ± 1.67
**NeAnd 20**	122.3 ± 1.7	129.3 ± 0.2	0.192 ± 0.035	0.204 ± 0.009	-28.96 ± 0.72	-29.40 ± 3.01
**NeAnd 40**	119.1 ± 0.9	138.2 ± 2.5	0.139 ± 0.025	0.200 ± 0.004	-28.68 ± 1.54	-29.94 ± 0.57
**NeAndG 5**	147.6 ± 1.4	152.9 ± 1.4	0.207 ± 0.010	0.200 ± 0.026	-61.68 ± 2.95	-69.75 ± 17.75
**NeAndG 10**	132.9 ± 2.8	146.2 ± 1.7	0.158 ± 0.018	0.236 ± 0.025	-63.87 ± 4.76	-71.54 ± 4.53
**NeAndG 20**	135.8 ± 0.1	142.9 ± 2.2	0.179 ± 0.008	0.161 ± 0.037	-68.43 ± 5.29	-63.97 ± 5.97

NeAnd 10, 20, and 40 – nanoemulsion with 10, 20, and 40 mg/mL of andiroba oil, respectively; NeAndG 5, 10, and 20 – nanoemulsion incorporated into a gel matrix with a final concentration of andiroba oil of 5, 10, and 20 mg/mL respectively. The pH of the formulations diluted at a ratio of 1:100 (v/v) in deionized water for dynamic light scattering (DLS), and electrophoretic light scattering (ELS)analysis was 7.

Supplementary Table 2. FTIR wavenumber (cm-1) of compounds and NeAnd.

	**Wavenumber (cm^{-1})**		
**Assignment**	**Surfactants**	**Andiroba Oil**	**NeAnd**
ν_s (O-H)	3329	-	3358
ν_{as} ($-CH_3$)	3002	3003	3006
ν_s ($-CH_3$)	2958	2951	2953
ν_{as} ($-CH_2$)	2920	2920	2920
ν_s ($-CH_2$)	2851	2851	2850
ν_s (C=O)	1730	1742	1720 - 1729

ν_s (C=C)	1650	-	1650
δ_{as} ($N^+(CH_3)$)	1663	-	1456
sc (CH_2)	-	1459	1490
δ ($-CH_3$)	1383	1374	1379
ν_{as} (PO^{2-})	1230	-	1251
ν_s (PO^{2-})	1174	-	1161
ν_s (C-O)	-	1159	1231
ν_s (CO-O-C)	1090	-	1092
ν_{as} (CO-O-C)	1060	-	1061
ν_{as} ($N^+(CH_3)$)	968	-	967
ν_s ($N^+(CH_3)$)	953	-	925
ν_{as} (PO^-)	822	-	823
δ ($-CH_2$)	719	722	719

Supplementary Table 3. FTIR wavenumber (cm^{-1}) of NeAnd, Carbopol Gel and NeAndG.

	Wavenumber (cm^{-1})		
**Assignment**	**NeAnd**	**Carbopol Gel Base**	**NeAndG**
ν_s $(O-H)_{broand}$	3358	3207	3300
ν_{as} ($-CH_3$)	3006	-	3007
ν_s ($-CH_3$)	2953	-	2956
ν_{as} ($-CH_2$)	2920	2936	2920
ν_s ($-CH_2$)	2850	2871	2851
ν_s (C=O)	1720 e 1729	1665	1736
ν_s (C=C)	1650	-	1666
ν_{as} (C-O)	-	1556	1558
δ (O-H)	-	1551	1454
δ_{as} ($N^+(CH_3)$)	1456	-	-
sc (CH_2)	1490	-	-
δ ($-CH_3$)	1379	-	-
ν_s (C-N)	-	1325	1358
ν_s $(C-O)_{alcool}$	-	1093	1069
ν_{as} (PO^{2-})	1251	-	1228
ν_s (PO^{2-})	1161	-	1167
ν_s (C-O)	1231	1393	1454
ν_s (CO-O-C)	1092	-	1093
ν_{as} (CO-O-C)	1061	-	-

ν_{as} ($N^+(CH_3)$)	967	-	967
ν_s ($N^+(CH_3)$)	925	-	917
ν_{as} (PO^-)	823	-	811
δ (C-H)	-	847	
δ ($-CH_2$)	719	-	724
